# The Risk Screener Violence (RS-V): retrospective prediction of violent and aggressive incidents within the prison setting

**DOI:** 10.3389/fpsyg.2024.1359535

**Published:** 2024-03-14

**Authors:** Marjam V. Smeekens, Michiel De Vries Robbé, Arne Popma, Maaike M. Kempes

**Affiliations:** ^1^The Netherlands Institute for Forensic Psychiatry and Psychology, Utrecht, Netherlands; ^2^Institute of Education and Child Studies, Faculty of Social and Behavioural Sciences, Leiden University, Leiden, Netherlands; ^3^Child and Adolescent Psychiatry and Psychosocial Care, Amsterdam University Medical Center (UMC), Vrije Universiteit (VU) University Medical Center, Amsterdam, Netherlands; ^4^Department of Psychiatry and Behavioural Neurosciences, McMaster University, Hamilton, ON, Canada

**Keywords:** risk screening, violence risk, prison, institutional violence, aggression

## Abstract

**Introduction:**

Physical and verbal violence toward staff or other detained individuals is a reoccurring problem within correctional facilities. Screening for violence risk within the prison setting could provide a valuable first step in the prevention of institutional violence. The brief and compact Risk Screener Violence (RS-V) has shown to be an efficient new method for assessing concerns regarding post-release violent offending for incarcerated persons. This study aimed to find out whether the RS-V is also able to predict future violent and aggressive incidents during imprisonment.

**Methods:**

The predictive validity of the RS-V for future violent and aggressive incidents during a follow-up time of 4 months within prison was analyzed, using a file-based design. Violent incidents toward staff and other inmates (physical violence and violent threats), other aggressive incidents (aggression toward objects and verbal disruptive behavior), and both categories combined, were included as outcome measures based on disciplinary reports.

**Results:**

The RS-V showed medium to large predictive values for both violent and aggressive behavior during prison stay. In particular, good predictive values of the RS-V were found for violence toward prison staff.

**Discussion:**

This study shows that, besides post-release violent recidivism, the RS-V is able to accurately predict future violent and aggressive incidents during prison stay. By correctly differentiating between low concern and high concern individuals, the RS-V aims to contribute to more personalized interventions and risk management and, subsequently, to improved prison safety. Future studies using prospective prison practice data are needed to further support the validity of the RS-V regarding institutional violence.

## 1 Introduction

The incidence of violent behavior within prison settings has, besides disrupting the social/ward climate, major psychological and emotional consequences for detained individuals and personnel (Wooldredge, 1991). For instance, prison employees that experience prison violence are more likely to develop post-traumatic stress disorder (PTSD) or burn-out (Boudoukha et al., 2013; Lerman et al., 2022). Similar consequences apply for incarcerated individuals. Namely, victimization may diminish the level of safety inmates experience (Wolff and Shi, 2009), and appears to be associated with emotional distress (e.g., depressive or anxious symptoms), PTSD, and anti-social behavior (Hochstetler et al., 2004; Boxer et al., 2009). Even witnessing victimization may have similar adverse effects (Boxer et al., 2009; Daquin et al., 2016). Preventing the occurrence of institutional violent incidents is therefore of great importance.

Regarding the prevalence of in-prison violence, self-report data from the U.S. shows that 13%–35% of the incarcerated individuals reported prisoner-to-prisoner physical assault, and 10%–32% reported prisoner-to-staff physical assault within a time-frame of 6 months (Wolff et al., 2007). Furthermore, in 2020, a total of 24,617 reports of misconduct were made within the Dutch prison system, which roughly houses 35,000 individuals a year (Dekker, 2021). In total, 3% of these reports mentioned threats toward staff and in 2% of the cases, there was an occurrence of physical violence toward staff. It has been suggested that the actual rate of violent behavior within prisons is likely even higher (Byrne and Hummer, 2007). More importantly, every violent/aggressive incident is one too many.

Two prominent theories within prison practice try to explain what causes detained individuals to act out in violent behavior. First, deprivation models suggest that the prison environment itself generates stress and frustration among inmates (Sykes, 2007). More specifically, prison specific factors cause detained individuals to develop negative attitudes toward the prison system, which in turn could lead to prison misconduct (Jiang and Fisher-Giorlando, 2002). Examples of these “pains of imprisonment” are the loss of autonomy and security, and the pain of being confined and deprived from liberty (Jiang and Fisher-Giorlando, 2002; Vogelvang et al., 2016).

On the contrary, importation theories highlight that misconduct within prisons is caused by individual pre-prison factors (Irwin and Cressey, 1962). Meaning that unique personal and behavioral characteristics of an inmate influence his/her adjustment to prison life. These characteristics are already present before institutionalization, such as acquired skills, thinking patterns, impulsivity, trauma, and previous offending (Vogelvang et al., 2016). Individual characteristics determine the level of adjustment to the prison environment and whether an individual may, or may not, act violently in reaction to inevitable tensions that occur within the prison system. Although criticized, both deprivation theories and importation theories seem to explain variance within prison misconduct and should be viewed as complementary (Paterline and Petersen, 1999). Finally, situational factors (e.g., location, time and interactions with other detained individuals) are also viewed as relevant for explaining the occurrence of prison misconduct (Steinke, 1991; Jiang and Fisher-Giorlando, 2002).

Within the framework of the importation theories, several individual characteristics have proven to be associated with violent incidents during imprisonment. These are, for instance, previous violent behavior, drug and alcohol use, impulsivity and low self-control (Gendreau et al., 1997; Jiang and Fisher-Giorlando, 2002; DeLisi et al., 2010; Arbach-Lucioni et al., 2012). Other factors, such as motivation, social network support and aging have proven to be associated with a decrease of violent misconduct and may serve as a protective factor (Velarde, 2001; Van der Laan and Eichelsheim, 2013; Lai, 2019; Klepfisz et al., 2022). These risk and protective factors are often incorporated into risk assessment instruments. Risk assessment instruments are designed to estimate the risk of future (violent) offending and to eventually, if personalized interventions are implemented based on the observed risk level of an individual, help prevent violent behavior. Using validated instruments within the prison setting is important, since false negative predictions may potentially harm societal and prison safety (Kang and Wu, 2022). For instance, when individuals with a high violence risk are granted unjustified leaves. At the same time, false positive predictions may have an unnecessary negative impact on prisoner mental health, due to overly restrictive risk management. From a deprivation perspective, this may in turn lead to an increased risk of violence. There is an ongoing debate about the ideal cost ratio of false positives (potential harm to the individual) versus false negatives (potential harm to others), which is also context dependent (Rice and Harris, 2005; Kang and Wu, 2022). Although criminal justice professionals and the general public opinion seem to prefer risk assessment models with a higher rate of false positives (Netter, 2007; Barnes and Hyatt, 2012; Kang and Wu, 2022).

Several well-known risk assessment instruments, such as the HCR-20^*V*3^, the LS/CMI, the SAPROF, and the VRS have been validated and proven to be successful in predicting violent incidents within institutional settings (e.g., Belfrage et al., 2000; De Vries Robbé et al., 2016; Persson et al., 2017; Hogan and Olver, 2018; Abbiati et al., 2019). However, conducting extensive risk assessment with these instruments for all detained individuals is not always realistic due to the often limited behavioral expertise among the majority of prison employees, as well as constraints in time and resources (factors that are, in part, a result of prison management) (Russo et al., 2020). Because only a subgroup of detained individuals is at high risk of acting out in violent behavior during imprisonment, it is also not necessary or efficient to administer comprehensive risk assessment tools for every individual. These issues regarding the administration of extensive risk assessment for all individuals also existed within the Dutch prison setting. As a result, structured in-prison risk evaluation was only conducted for a small group of incarcerated individuals (e.g., for individuals with severe psychiatric problems who reside within specialized prison units or for individuals with serious transgressive behavior). However, in recent years, improving risk evaluation and management for all detained individuals became a top priority within the Dutch prison setting (Dutch Custodial Institutions Agency, 2021). Therefore, the Dutch prison system explored options for implementing a compact and brief violence risk screening tool that is more suitable for wide scale consistent use for the larger prison population.

A few risk screening instruments have been developed that can also be applied within correctional settings, such as the screening version of the LSI-R (Andrews and Bonta, 2001), the DASA (Ogloff and Daffern, 2006), and the screener version of the VRS (Wong and Gordon, 2007). However, these instruments lack important attributes that were deemed highly relevant within Dutch prisons, such as the explicit incorporation of both risks and strengths for violent behavior. Within the prison system in the Netherlands, there was a desire and a need to develop a new risk screening instrument which contains both risk and protective factors, is scored based on Structured Professional Judgment (SPJ) assumptions, focuses specifically on the prediction and prevention of violent behavior, includes a multidisciplinary consensus conclusion regarding the results of the screening, and can be conducted by prison employees without specific behavioral expertise (i.e., prison workers who are not psychiatrists or psychologists). This led to the development of the new Risk Screener Violence (RS-V) (De Vries Robbé and Van den End, 2020). The RS-V has been implemented in all Dutch prisons in 2021 for the violence risk screening of every individual admitted to prison.

Research shows that a relatively small part of the prison population is responsible for a relatively large part of (violent) incidents within prison (Duwe, 2020). If, by administering the RS-V, an improvement can be made within the early differentiation between individuals for whom there are high concern versus low concern individuals, accordingly, risk management can be allocated more efficiently and effectively, and as a result new violent incidents are more likely to be prevented. Furthermore, by serving as triage for administering further in-depth risk assessment (generally carried out during a later phase of detention) and by possibly improving the application of personalized rehabilitative interventions, the RS-V might be able to contribute to an increase in internal prison safety.

The current study aims to find out whether the RS-V is able to predict future violent incidents during imprisonment. More specifically, this study focuses on the predictive value of the RS-V for violent incidents (actual physical violence and violent threats) toward staff or fellow inmates, during a follow-up period of 4 months after the screening. Additionally, other aggressive incidents (aggression toward objects and verbal disruptive behavior), are included as an extra outcome measure to serve as a wider proxy of actual violent behavior. Furthermore, a third outcome measure is included, which comprises the combination of both outcome categories, violent and aggressive incidents.

The RS-V data included in this study are retrospective, meaning that the RS-V’s are scored by researchers based on file information of detainees from their prison records. Since a previous file-based pilot study on the RS-V has shown promising results regarding its psychometric properties for use during prison stay (De Vries Robbé et al., 2021), we expect sound predictive validity regarding institutional violence (toward staff as well as fellow inmates). Moreover, it is anticipated that aggressive behavior and both categories combined can also be predicted significantly by the RS-V. Further support for this hypothesis stems from the recent finding that the RS-V was scored with excellent interrater reliability and demonstrated successful in predicting violent offending after release (Smeekens et al., 2024), and thus was able to predict actual violent behavior.

## 2 Materials and methods

### 2.1 Participants

The sample of incarcerated persons that was selected for this study, was derived from an earlier retrospective study that looked into the predictive validity of the RS-V for post-release violent offending (Smeekens et al., 2024). The RS-V’s included by Smeekens et al. (2024) were rated by researchers based on prison reports regarding behavior during the last few months of detention, prior to the moment of discharge. These RS-V’s were then linked to violent offending after release. The group of detained individuals included by Smeekens et al. (2024) needed to adhere to several inclusion criteria. First, individuals needed to be released from a Dutch prison between September 2014 and September 2017, and they needed to have been formally convicted of the crime for which they received the corresponding prison sentence. Second, eligible individuals needed to have sufficient file information to retrospectively rate all parts of the RS-V. In addition, after release, participants needed to have remained within the Netherlands, stayed out of prison or a forensic clinic for at least half of the follow-up period, and not have passed away. The final sample of the study consisted of 571 individuals from 25 Dutch prisons (Smeekens et al., 2024).

For the current study, files were drawn from the previous study by determining which of the 571 incarcerated individuals had spent sufficient time in prison for the researchers to also be able to rate a RS-V shortly after admission. A detention period of at least 2 months was deemed necessary to retrospectively gather enough digital file information about each detainee in order to be able to reliably score the RS-V. Subsequently, 4 months of further prison stay was needed for the follow-up period (see section “2.3 Violent and aggressive incidents within prison”). Thus, an individual needed to have a total detention period of at least 6 months in order to be included within this study.

In total, 256 individuals adhered to this inclusion criterion. Then, 75 participants were excluded because more than two factors of the RS-V were indicated as “unknown” during the screening; these cases did not have sufficient file information to retrospectively score the RS-V after admission. Ultimately, 181 individuals (145 males, 36 females) adhered to all the inclusion criteria and were included in the current study. They had a mean age of 35 years (SD = 11.17, range = 18–66) and the mean duration of their detention period was 385 days (SD = 204.90, range = 179–1,215). The sample included 46 first-time detainees. See Table 1 for more information about previous violent behavior among this sample.

**TABLE 1:** The number of incarcerated individuals (n = 181) who demonstrated 1) previous convictions for interpersonal violence outside prison (in the community), and 2) previous disciplinary reports regarding interpersonal violence inside prison (during current or prior detentions).

	Historical risk factors	
**Frequency of conviction or disciplinary report**	**Item H1 of the RS-V: previous interpersonal violence (convictions) outside prison** **(*n*, %)**	**Item H2 of the RS-V: previous interpersonal violence (disciplinary reports) inside prison (*n*, %)**
0	43 (23.8)	115 (63.5)
1	23 (12.7)	32 (17.7)
2–3	39 (21.5)	21 (11.6)
4–5	20 (11.0)	6 (3.3)
>6	56 (30.9)	7 (3.9)

### 2.2 The Risk Screener Violence

The RS-V is a risk screening instrument, initially developed for prison settings, that aims to estimate concerns for future violent behavior of an individual (De Vries Robbé and Van den End, 2020). The RS-V offers a first general impression regarding the most important risk factors and protective factors of each individual. The following definition of violent behavior is used within the RS-V: attempting, threatening with, or actually showing physical violence toward others (including sexual violence). The RS-V may be used within different custody levels and is scored with excellent inter-rater reliability and good predictive validity regarding post-release violent offending for males and females (based on retrospective data) (Smeekens et al., 2024). Within prison practice, the RS-V is administered for every individual during the first 6 (in some prisons 9) weeks of incarceration and is administered again later on during detention when a detainee qualifies for leaves. In addition, the RS-V may be reassessed intermediately at any time whenever deemed useful (e.g., when a considerable amount of time has passed since the last screening, when a severely aggressive incident has occurred, or when new risk-related information has become available). Within this retrospective file study, RS-V’s were rated based on behavioral reports regarding the first few months of imprisonment (see section “2.4 Procedure”). The RS-V is more compact than extensive risk assessment instruments and consists of 10 factors and 3 final conclusions divided over 3 parts.

The first part of the RS-V consists of two historical risk factors. These are “previous interpersonal violence outside prison” (H1), and “previous interpersonal violence inside prison” (H2). Both factors are scored on a five-point scale (0–4) based on the frequency of the respective behavior within the entire past of the individual (see Table 1). H1 is rated based on actual convictions within the official criminal record of an individual and H2 is rated based on disciplinary reports within the digital prison file of a detainee. As can be seen in Table 2, the second part of the RS-V contains four dynamic risk factors (R1 to R4) and four dynamic protective factors (P1 to P4). These factors are scored on a three-point scale: 0 = “not or hardly present,” 1 = “moderately present,” or 2 = “clearly present.” A higher score indicates the presence of a problem (risk factor) or a strength (protective factor). These dynamic factors are rated based on the behavioral observations of prison employees during the months prior to screening (since admission, or the past 6 months of prison stay). More specifically, digital records of, for example, urine test results and disciplinary reports are consulted, as well as reports from case managers, prison officers, nurses, and other prison staff. The rating of an item is to be supported by sound argumentation, described by the assessor on the rating form. If relevant, additional case-specific historical and dynamic information related to the individual’s violence risk may be added.

**TABLE 2:** The historical risk factors, dynamic risk factors, dynamic protective factors, and final conclusions included in the RS-V.

Part 1. Historical risk factors
H1. Previous interpersonal violence outside prison
H2. Previous interpersonal violence inside prison
**Part 2. Dynamic factors (past 6 months in prison)**
**Risk factors**
R1. Recent interpersonal violence
R2. Substance use
R3. Negative/defiant attitude
R4. Impulsive behavior
**Protective factors**
P1. Following rules and agreements
P2. Coping with problems and frustrations
P3. Positive influences from social network
P4. Motivation for crime free future
**Part 3. Final conclusions (coming 6 months)**
**Concerns regarding future**
A. Violence inside prison
B. Violence outside prison after release
C. Violence outside prison during leaves

The third part of the RS-V consists of three final conclusions. Within prison practice, these final conclusions are formulated during a multidisciplinary team meeting. Whereas in the current study, the conclusions were made by a single researcher that rated the RS-V. The three final conclusions are formulated based on the findings documented by the rater in part one (historical factors) and part two (dynamic factors) of the RS-V. The three final conclusions express concerns about future interpersonal violence regarding the following 6 months in an SPJ manner. This means that the RS-V aims to be valid for a period of 6 months. The final conclusions consider concerns regarding the risk of (A) in-prison violence, (B) post-release violence, and (C) violence during leaves from prison (only rated in case of proposed leaves during prison stay). They are rated as: 0 = “low concerned”; 1 = “moderate concerned”; or 2 = “serious concerned.” Table 2 shows the factors and final conclusions that are included in the RS-V. A factor can be scored as “unknown” if there is not enough information available for a reliable rating. When more than two factors in part one or part two are scored as unknown, the third part of the RS-V cannot be completed and the RS-V is considered invalid.

During the multidisciplinary team meetings in prison practice, possible follow-up measures are discussed for individuals for whom there are moderate or serious concerns regarding future violent behavior. Examples of these follow-up measures are: single-celling, contacting the prison psychologist regarding specific concerning observations, offering targeted behavioral interventions such as anger management training or addiction treatment, conducting extensive risk assessment (e.g., by means of the HCR-20^*V*3^ and the SAPROF), informing decision-making regarding prison leaves or other privileges, and discussing the RS-V results with the incarcerated person and/or with other professionals both inside and outside prison. For more information on the RS-V, see the study of Smeekens et al. (2024).

### 2.3 Violent and aggressive incidents within prison

Since the RS-V has been developed to specifically predict violent behavior, this study included outcome measures that describe violent behavior or proxies thereof. Other types of misconduct during prison stay, such as positive urine tests or possession of contraband, were not included as outcome measures. The three dichotomous outcome measures included in this study were: (1) violent incidents: actual physical violence and violent threats toward other people; (2) aggressive incidents: aggression toward objects and verbal disruptive behavior; and (3) both the categories of violent incidents and aggressive incidents combined. Aggression toward objects was defined as aggressive behavior (such as slamming or kicking) toward objects, such as walls, doors, or trashcans. Verbal disruptive behavior was specified as verbally abusing, insulting, offending or challenging other detained individuals or staff, without explicit violent threats. Regarding violent incidents (physical violence or verbal threats), a distinction was made between the type of victim (other detained individuals or personnel) the aggression was directed at, for aggressive incidents it was not possible to make this distinction.

The violent and aggressive incidents were scored as 0 (no/not present) or 1 (yes/present) for each individual, within a timeframe of 4 months after rating the RS-V. Although some individuals showed multiple incidents of the same type during follow-up, these were counted as 1 (yes). Even though the final conclusions within the third part of the RS-V make predictions about the following 6 months, a follow-up period of 4 months was used for this study. A 4-month-follow-up period was deemed sufficiently long to be able to detect violent behavior, yet still ensuring a relatively large sample size, given that the majority of Dutch detainees is already released within 6 months.

The occurrence of violent and aggressive incidents (yes/no) during the follow-up per individual was scored based on reports within the central digital prison archive of the Dutch Ministry of Justice and Security, where researchers had access to the prison file information of each included detainee. Specifically, records regarding disciplinary write-ups and disciplinary decisions/measures were consulted. Table 3 shows the occurrence of violent and other aggressive incidents among the included sample of detained individuals. It also includes information regarding the victim of the violent behavior.

**TABLE 3:** The occurrence rates (yes/no) of the different types of incidents within prison during a 4-month-follow-up period.

Incident category	Number of detained individuals (*n* = 181)	%
**Violent incidents**	18	9.9
Toward staff	10	5.5
Physical violence	5	2.8
Violent threats	6	3.3
Toward other detained individuals	9	5.0
Physical violence	8	4.4
Violent threats	2	1.1
**Aggressive incidents**	23	12.7
Aggression toward objects	8	4.4
Verbal disruptive behavior	16	8.8
**Any violent or aggressive incident**	30	16.6

Results between incident categories may overlap. Meaning that an individual could have committed incidents within different categories and that subcategories will not add up to the total.

### 2.4 Procedure

The study protocol for this study was approved by the Ethics Committee of the Institute of Pedagogical Science of the University of Leiden (Reference Number: ECPW-2021/33). Data collection for this retrospective file-based study took place between January 2022 and December 2022. Within prison practice, the RS-V is completed by employees in prison. In the current study, the rating was done by researchers and graduate students (*n* = 4) trained in using the RS-V and the prison records.

The data collection consisted of three steps. The first step was to check whether an individual adhered to the inclusion criteria (see section “2.1 Participants”). If more than two factors of the RS-V were scored as unknown within 2 months after admission, the researcher broadened the scope of the search within the digital file of the detainee by adding an extra month of prison documentation. Subsequently, the researcher checked again whether there was sufficient file information available. This process was repeated until there was enough information available to rate the RS-V. This process continued until a maximum of 5 months after admission. If, after 5 months of imprisonment, an individual still did not have enough available file information, the particular case was excluded from the dataset.

The second step of data collection consisted of scoring the RS-V’s for all the included incarcerated persons. In order to prevent bias when scoring the RS-V, the process of including an individual and the scoring of the RS-V was divided between the researchers. The first researcher checked the inclusion criteria and scored item H1. Then, the second researcher scored item H2, items R1 to R4, items P1 to P4, and possibly additional historical and dynamic information. The dynamic factors were rated based on prison reports written during the beginning of the detention period: from admission until 2, 3, 4, or 5 months after admission (depending on the availability of file information, see above). The second researcher also scored part three of the RS-V: the final conclusions, based on the ratings on all factors (including H1) and the additional historical and dynamic information. However, since the outcome measures of the current study concern in-prison violence and aggression, we only included final conclusion A (concerns regarding violence inside prison) in our data-analyses.

The third and final step of data collection was to score the outcome measures, violent and other types of aggressive incidents, based on incident reports within the digital file of each detainee. First, the correct timeframe was selected within the digital file: from the date of rating part three of the RS-V until 4 months later. The incident reports within that timeframe were then scored using a scoring form that included the date, type of incident, and, if applicable, at whom the violent incident was directed (prison staff or fellow detained individuals). While rating the RS-V and the final conclusions of the RS-V in step one and step two, raters were blind to the outcomes collected in step three.

### 2.5 Statistical design

The data were analyzed using IBM SPSS Statistics version 27. Missing values were replaced through pro-rating: each missing value received the mean score on the corresponding subscale for the individual case. On average, 1.4 factors were missing per incarcerated person. The protective factor P3 (positive influences from social network) had the largest number of missing values, namely 75.7%.^1^ This shows that, within the prison records, the availability of information about the social network of an individual within the prison records was often not sufficient.

Within prison practice, the result of the screening is comprised of the final conclusions regarding concerns about future violence. However, for the purpose of the present empirical study, the ratings of the individual factors were added up into subscale scores and a total score. In order to be able to do so, the historical factors were transformed from a five-point rating scale to a three-point rating scale. The ratings 1–2 were changed into a score of 1 and ratings 3–4 into a score of 2. Subsequently, the historical and dynamic risk factors were added up, while subtracting the dynamic protective factors, to arrive at an overall total score of risk corrected for protection. A more negative total score on the RS-V indicates a greater presence of protective factors in comparison to risk factors. The adjusted subscale scores and total score were used in further analyses.

Descriptive analyses of the unadjusted individual items, the adjusted subscale scores, the adjusted RS-V total score and the final conclusion A (concerns regarding violence inside prison) were conducted. Since for many detained persons protective factors were rated as more present than risk factors, the average total score resulted in a negative value of −1.92 (SD = 4.45, range = −8 to 11.60) (see Table 4).

**TABLE 4:** The descriptive statistics (*M*, SD, Min., and Max.) of the separate items, the subscale scores, total score, and final conclusion A of the RS-V for the total sample (*n* = 181).

	*M*	SD	Min.	Max.
H1	2.13	1.56	0	4
H2	0.66	1.06	0	4
R1	0.10	0.34	0	2
R2	0.60	0.68	0	2
R3	0.40	0.66	0	2
R4	0.52	0.79	0	2
P1	1.50	0.71	0	2
P2	1.39	0.73	0	2
P3	1.18	0.82	0	2
P4	0.99	0.78	0	2
Historical risk factors	1.67	1.38	0	4.80
Dynamic risk factors	1.51	1.74	0	8
Dynamic protective factors	5.11	2.19	0	8
RS-V total score	−1.92	4.45	−8	11.60
Final conclusion A: concerns regarding violence inside prison	0.48	0.70	0	2

The descriptive statistics of the individual factors are the unadjusted scores and the descriptive statistics of the subscales and total score are the adjusted scores (see section “2.5 Statistical design”).

In order to investigate the predictive validity of the RS-V for violent and aggressive incidents, receiver operating curve (ROC) analyses were conducted. ROC analyses result in area under the curve (AUC) values, which, in this case, represent the ability for the RS-V to correctly identify whether an individual will commit future violent or aggressive incidents during the follow-up period in prison. AUC values can be classified as small (between 0.56 and 0.64), medium (between 0.64 and 0.71), or large (above 0.71) (Rice and Harris, 2005). For instance, an AUC value of 0.749 means that there is a probability of 75% that a randomly selected violent individual will have a higher score on the RS-V than a randomly selected non-violent individual. Regarding the subscale of the dynamic protective factors, the AUC values were mirrored, indicating that higher AUC values reflect a protective effect against the occurrence of incidents. ROC analyses were conducted for violent incidents, aggressive incidents, and both of these incident categories combined. In addition, the category of violent incidents was further divided into violence toward staff and violence toward other detained persons, for which two separate further ROC analyses were conducted.

## 3 Results

### 3.1 Descriptive statistics

Table 4 shows the means and standard deviations of the RS-V on the separate items, the subscale scores, total score, and the final conclusion A. Overall, the dynamic protective factors were rated relatively high compared to the dynamic risk factors. In total, for 65% of the detained individuals included in the current study, the researchers were “low concerned” regarding future violent behavior inside prison. While 23% of the detainees’ final conclusion A was rated as “moderate concerned,” and 12% as “serious concerned.”

### 3.2 Predictive validity of the RS-V for violent and aggressive incidents within prison

The true positives, true negatives, false positives, and false negatives regarding the occurrence of violent incidents within prison can be found in Table 5. This table shows that, based on final conclusion A, there are relatively more false positive predictions than false negative predictions regarding violent incidents within prison during the 4-month-follow-up period. Additionally, 4.3% of the individuals with low concerns committed a violent incident, for the group with moderate concerns this was 14.3%, and for the group with serious concerns this was 31.8%.

**TABLE 5:** The concerns expressed within final conclusion A (concerns regarding violence inside prison) in contrast to the actual occurrence of violent incidents during a 4-month-follow-up period within prison.

Final conclusion A: concerns regarding violence inside prison	No violent incident (*n*, % of total)	Violent incident (*n*, % of total)	Total (*n*, % of total)
Low concerns	112 (61.9)	5 (2.8)	117 (64.6)
Moderate concerns	36 (19.9)	6 (3.3)	42 (23.2)
Serious concerns	15 (8.3)	7 (3.9)	22 (12.2)
Total	163 (90.1)	18 (9.9)	181 (100)

Table 6 displays the AUC values of the RS-V regarding the prediction of violent and aggressive incidents. Most of the subscale scores, the total RS-V score and the final conclusion A (concerns regarding violence inside prison) were significant predictors of violent incidents, aggressive incident, and both incidents categories combined. The significant AUC values were moderate to large (AUC = 0.664–0.759). Only the subscale score of the historical risk factors was a non-significant predictor of violent incidents. The same result was found for the smaller category of violence toward staff: all factors except the historical risk factors were significant predictors of violent incidents toward personnel. However, none of the subscales of the RS-V were significant predictors for violence toward other detained persons specifically.

**TABLE 6:** The area under the curve (AUC) and 95% confidence intervals (CI) of the subscale scores, total score, and final conclusion A (concerns regarding violence inside prison) of the RS-V for different types of incidents regarding 4 months follow-up during imprisonment.

	Historical risk factors		Dynamic risk factors		Dynamic protective factors		RS-V total score		Final conclusion A: concerns regarding violence inside prison	
	**AUC**	**95% CI**	**AUC**	**95% CI**	**AUC**	**95% CI**	**AUC**	**95% CI**	**AUC**	**95% CI**
Violent incidents (prevalence = 10%)	0.604	(0.46, 0.75)	0.749***	(0.64, 0.86)	0.664*	(0.53, 0.80)	0.711**	(0.58, 0.84)	0.732***	(0.60, 0.86)
Toward staff (prevalence = 6%)	0.670	(0.62, 0.94)	0.808***	(0.71, 0.91)	0.694*	(0.55, 0.84)	0.781**	(0.68, 0.89)	0.778**	(0.62, 0.94)
Toward other detained individuals (prevalence = 5%)	0.531	(0.33, 0.73)	0.687	(0.50, 0.88)	0.646	(0.44, 0.85)	0.642	(0.42, 0.86)	0.684	(0.50, 0.87)
Aggressive incidents (prevalence = 13%)	0.686**	(0.57, 0.80)	0.681**	(0.56, 0.80)	0.686**	(0.58, 0.79)	0.711***	(0.60, 0.83)	0.718***	(0.60, 0.84)
Violent incidents and aggressive incidents (prevalence = 17%)	0.684***	(0.58, 0.79)	0.733***	(0.63, 0.83)	0.697***	(0.60, 0.79)	0.744***	(0.65, 0.84)	0.759***	(0.66, 0.86)

*p < 0.05

**p < 0.01

***p < 0.001.

## 4 Discussion

This study aimed to find out whether a newly developed risk screening instrument, called the RS-V, is able to predict in-prison violent and aggressive incidents. As such, this study contributes to the further validation of the RS-V based on retrospective data. A previous study found promising results for the RS-V regarding the prediction of post-release violent offending (Smeekens et al., 2024). The current study reveals that the RS-V is also able to adequately predict violent behavior within the prison setting at 4-months follow-up. More specifically, the predictive validity of the RS-V for violent incidents (physical violence and violent threats toward others), aggressive incidents (aggression toward objects and verbal disruptive behavior), and both categories of incidents combined was large for the total score and the final concerns regarding in-prison violence. The AUC value of the RS-V total score (0.74) and final concerns (0.76) found within this study for the combined aggression outcome, are comparable to the AUC values related to more extensive risk assessment instruments that are used within Dutch forensic practice to predict institutional violence (e.g., total score HCR-20^V3^ = 0.77, total score SAPROF = 0.76, and overall final risk judgment = 0.74; De Vries Robbé et al., 2016). For the subscale scores, moderate to large predictive validities were found, apart from the historical subscale, which was not significant for the prediction of interpersonal in-prison violence. Overall, this study found sound predictive values for the RS-V for violent incidents toward prison staff, whereas none of the scales of the RS-V demonstrated significant predictive value for violence toward fellow detained individuals. However, this result should be interpreted carefully due to the relatively low base rate of incidents within this category.

The descriptive statistics show that, overall, the dynamic protective factors are rated relatively high compared to the dynamic risk factors. This seems to indicate that, on average, the majority of the prison sample included in this study behaved fairly well during the first months of their prison stay. Based on final conclusion A of the RS-V, only approximately 1 out of 10 participants received high concerns for future violent behavior within prison. Accordingly, the base rates of violent and aggressive incidents were also relatively low. Nevertheless, the RS-V was able to predict quite accurately which individuals would cause aggressive incidents and which ones would not. In addition, this study shows that the concerns expressed within final conclusion A lead to more false positive predictions than false negative predictions regarding violent incidents. This ratio is generally preferred for risk assessment tools (Netter, 2007; Barnes and Hyatt, 2012; Kang and Wu, 2022). Moreover, false positive predictions may not necessarily be problematic for the individual, as it could result in enhanced attention on ensuring a safe reintegration into society, accompanied by appropriate individualized interventions. The results of this study reveal that the subscale scores, total score and final conclusion A (concerns regarding violence inside prison) of the RS-V are almost all significant (medium to large) predictors for violent incidents toward others, aggressive incidents, and both incidents combined. This is in line with our hypothesis, previous findings regarding the RS-V, and previous research that shows that other risk screening instruments are also predictive of short-term institutional aggression (i.e., the DASA), and disciplinary infractions (i.e., the LSI-R:SV) (Walters and Schlauch, 2008; Chu et al., 2013; Griffith et al., 2013). These results therefore indicate that the use of risk screening instruments within the prison setting appears applicable.

Our study did not find specific predictive value of the historical risk factors subscale for violent incidents within prison. Although this result seems somewhat surprising, it could be explained by deprivation models, which suggest that being imprisoned and being deprived from freedom may cause stress and frustration among detained persons which could lead to them acting out in violent behavior (Jiang and Fisher-Giorlando, 2002; Sykes, 2007). In fact, looking more closely, it appears especially previous violence outside of the prison context (factor H1) has limited predictive value for behavior during imprisonment. It could be that specific situations, that are only present within the prison setting, cause people to be violent and act differently than when being in the community, where these specific situational factors are not or less present. Similarly, it could also be the case that the prison context in general has a protective effect and that specific triggers for committing violence, which are present outside of the prison context, are less present within the prison setting. These findings will have to be studied more closely prospectively within the prison context.

The importation models, on the other hand, may explain the sound predictive value of the dynamic subscale scores for future violent and aggressive misconduct in prison. The dynamic risk factors subscale consists of specific personal and behavioral characteristics, such as impulsive behavior and substance use, which are supposedly associated with an increase in violent behavior (Gendreau et al., 1997; Arbach-Lucioni et al., 2012). On the contrary, the dynamic protective factors subscale is comprised of personal characteristics that are potential protectors against violent misconduct, such as social network support and motivation (Van der Laan and Eichelsheim, 2013; Lai, 2019; Klepfisz et al., 2022). The presence or absence of individual dynamic factors largely determines whether an individual may act out in violent behavior within prison, as stated by the importation models.

It was expected that the RS-V would predict both violence toward staff and violence toward other incarcerated individuals. This retrospective study found that the RS-V was primarily predictive of the former. The behavioral reports included in the files of detainees, that are used to score the RS-V, are filled in by different prison employees (e.g., administrator, prison officer, case manager, and nurse). It could be that prison staff is not fully able to observe all the interactions that occur between prisoners, in contrast to aggression toward employees, resulting in so-called “dark numbers.” Meaning: violent and aggressive incidents among detained individuals that did occur may not be present within the digital prison records and therefore not reported in this study. This could influence the reliability of the documentation of violent incidents between prisoners. Especially when it comes to violent threats among incarcerated individuals, which had a base rate of only 1.1% (compared to 3.3% toward staff). This study did not investigate the quality and quantity of the reports within the prison records. In general, the results regarding differences between staff and fellow inmate violence should be interpreted with caution due to the relatively low base rate in each group separately. In order to draw more firm conclusions regarding the predictive value of the RS-V for violence toward staff versus violence toward other detained individuals, this study should be replicated with a larger sample size. A prospective study that is currently being conducted, analyzing a large number of RS-V’s filled in by prison employees, may give more insight into this distinction.

### 4.1 Limitations

Despite carefully conducting this study, some limitations need to be mentioned. The first limitation concerns the availability of information within the digital prison records. Within this study, there was a relatively high number of cases that were excluded due to a limited amount of file information during the first few months of imprisonment (72 of 255 cases, 28%). This can be explained by the retrospective design, since the RS-V’s included in this study were scored by researchers and not by prison employees, the scoring of the RS-V depended on the quality and quantity of the reports that were available within the file of a detainee. Moreover, these files concerned data from before the implementation of the RS-V within Dutch prison practice. It would be expected that since the implementation of the RS-V, record keeping regarding specific risk and protective factors has improved significantly. Future prospective studies in prison practice will be able to investigate this assumption. In addition, scoring the occurrence of violent and aggressive incidents also depended on the completeness of information within the digital central archive. Furthermore, although the researchers were well trained in the use of the RS-V, they did not have first-hand experience working with incarcerated individuals in the prison setting. This may have dampened the predictive validity findings regarding the final conclusions. Using prospective data (actual RS-V’s from prison practice) will possibly overcome this problem because of the richness of information about each individual.

Another limitation of this study is that the RS-V’s were scored based on reports during the first few months of imprisonment. It could be that individuals may show different behavior during the first weeks of imprisonment than in a later phase of detention. Possible reasons as for why these behavioral differences may occur could be that an individual is experiencing withdrawal symptoms from an addiction, the new prison environment may be stressful, or the individual simply needs to get used to the prison setting. It is therefore recommended to routinely score the RS-V of an individual again over time to gain insight into possible changes in dynamic risk and protective factors, in order to be able to accurately re-evaluate concerns regarding violent behavior periodically.

A final limitation of this study is the included sample of relatively long-term detainees. Even though the follow-up period for detecting violent behavior in this study was shortened to 4 months instead of 6 months (which is the intended prediction time-frame of the RS-V, see section “2 Materials and methods”), this study still included a large number of relatively long-term detainees. Most detained individuals (69%) within the prison system in the Netherlands are released within 3 months (Dutch Custodial Institutions Agency, 2022). The individuals included in this study, with an average detention period of 385 days, are thus in reality a minority of the total prison population within the Netherlands. However, including individuals with a longer detention duration was necessary to score the RS-V and collect reliable outcome data (see section “2 Materials and methods”). In addition, since the RS-V is initially developed to make predictions about the following 6 months, future research could look into including longer follow-up periods to find out whether the RS-V is able to predict violent behavior within prison in the longer-term.

## 5 Conclusion

To summarize, this study contributes to the further validation of the RS-V by showing that the RS-V is not only able to predict future violent offending post-release but also future interpersonal violence and general aggression within the prison setting. By correctly differentiating between low concern and high concern individuals, the RS-V aims to contribute to the implementation of more tailored interventions and risk management and, subsequently, to a decrease in violent incidents and an increase in internal prison safety. Diminishing victimization and improving internal safety is an important goal within institutional settings, as this will most likely contribute to the prevention of the development of serious psychological problems, emotional distress, and/or further adverse and criminological outcomes among detained persons. Furthermore, it will improve the safety and wellbeing of prison staff and their overall work satisfaction. Implementing the RS-V as a global screening instrument could potentially be a valuable addition in achieving these goals. However, conducting prospective studies with RS-V’s that are rated by prison personnel are necessary to determine the robustness of the results of the current study.

An important implication for prison practice is to actively use the results of the RS-V and discuss how personalized interventions can be tailored to these concerns. The way prison staff responds to the observed concerns regarding in-prison violence is essential in the prevention of future violent incidents. For example, if an individual shows serious concerns regarding future violence, he/she might be in need of anger management training or, in case of forthcoming release, aftercare facilities need to be informed and in-depth comprehensive risk assessment may be advisable. Achieving an effective response of prison employees based on the results of the RS-V, may require the development of new expertise through risk management training and improved intervention initiatives within the prison setting. This is a challenging task for prison practice, but it seems well worth investing in when aiming to improve prison safety. The succession of the results of the RS-V requires ongoing attention and research within prison practice. Future studies could look into whether personalized interventions are actually applied and implemented in line with the final conclusions of the RS-V. Another important next step is to investigate RS-V’s that are filled in by prison employees. Since the RS-V has been implemented in all 25 Dutch prisons in 2021, it will be possible to prospectively analyze data regarding these RS-V’s from prison practice in future studies and compare the results between retrospective and prospective studies with the RS-V.

## Data availability statement

The datasets presented in this article are not readily available because the dataset contains highly sensitive and personal data. Requests to access the datasets should be directed to MS, m.smeekens@dji.minjus.nl.

## Ethics statement

The studies involving humans were approved by the Ethics Committee of the Institute of Pedagogical Science of the University of Leiden (Reference Number: ECPW-2021/33). The studies were conducted in accordance with the local legislation and institutional requirements. Written informed consent for participation was not required from the participants or the participants’ legal guardians/next of kin in accordance with the national legislation and institutional requirements.

## Author contributions

MS: Conceptualization, Formal analysis, Investigation, Methodology, Project administration, Visualization, Writing – original draft. MD: Conceptualization, Funding acquisition, Methodology, Supervision, Writing – review and editing. AP: Conceptualization, Supervision, Writing – review and editing. MK: Conceptualization, Funding acquisition, Methodology, Supervision, Writing – review and editing.

## Footnotes

1 Due to the large amount of missing values of this particular factor, all ROC analyses were additionally run without P3. This only affected the AUC values to a negligible extent (0.01). Therefore, the proposed pro-rated subscale scores and total score were deemed warranted.
